# A Myb Transcription Factor of *Phytophthora sojae*, Regulated by MAP Kinase PsSAK1, Is Required for Zoospore Development

**DOI:** 10.1371/journal.pone.0040246

**Published:** 2012-06-29

**Authors:** Meng Zhang, Jing Lu, Kai Tao, Wenwu Ye, Aining Li, Xiaoyun Liu, Liang Kong, Suomeng Dong, Xiaobo Zheng, Yuanchao Wang

**Affiliations:** Key Laboratory of Integrated Management of Crop Diseases and Pests, College of Plant Protection, Nanjing Agricultural University, Ministry of Education, Nanjing, China; Soonchunhyang University, Republic of Korea

## Abstract

*PsSAK1*, a mitogen-activated protein (MAP) kinase from *Phytophthora sojae*, plays an important role in host infection and zoospore viability. However, the downstream mechanism of PsSAK1 remains unclear. In this study, the 3'-tag digital gene expression (DGE) profiling method was applied to sequence the global transcriptional sequence of *PsSAK1*-silenced mutants during the cysts stage and 1.5 h after inoculation onto susceptible soybean leaf tissues. Compared with the gene expression levels of the recipient *P. sojae* strain, several candidates of Myb family were differentially expressed (up or down) in response to the loss of PsSAK1, including of a R2R3-type Myb transcription factor, *PsMYB1*. qRT-PCR indicated that the transcriptional level of *PsMYB1* decreased due to *PsSAK1* silencing. The transcriptional level of *PsMYB1* increased during sporulating hyphae, in germinated cysts, and early infection. Silencing of *PsMYB1* results in three phenotypes: a) no cleavage of the cytoplasm into uninucleate zoospores or release of normal zoospores, b) direct germination of sporangia, and c) afunction in zoospore-mediated plant infection. Our data indicate that the *PsMYB1* transcription factor functions downstream of MAP kinase *PsSAK1* and is required for zoospore development of *P. sojae*.

## Introduction

Oomycetes are economically important because they are destructive to crops and nursery stocks. Examples include *Phytophthora sojae* (causes soybean root rot), *Phytophthora infestans* (causes potato late blight), and *Phytophthora ramorum* (causes sudden oak death). Approximately 116 pathogenic *Phytophthora* species are currently known or described. New species, or new variants of known species, emerge continuously [1]. *P. sojae* is a soil-borne plant pathogen that causes soybean stem and root rot. Since its discovery in Indiana in 1948 and Ohio in 1951, Phytophthora rot has been frequently reported globally throughout most soybean-growing regions [2]. This disease may cause plant stand losses and complete yield reductions in susceptible soybean cultivars in poorly drained fields, costing millions of dollars each year. Therefore, slowing the assault of *P. sojae* pathogens is important.

*P. sojae* produce asexual sporangia which breed and release zoospores. Although in some species such as *P. infestans*, sporangia are released freely from aerial hyphae and serve as agents of dispersal, the sporangia of *P. sojae* are not readily released from the hyphae. Zoospores are the most important route of infection of roots, especially when the soil is flooded. In *P. sojae*, zoospores swim chemotactically toward compounds including the isoflavones daidzen and genistein released by roots of their host plants [3]. Due to their energy needs and fragility, asexual sporangia are ineffective resting structures. Instead the thick-walled and durable sexual spores, called oospores, can act as resting structures. As they remain viable between growing seasons, oospores are an important inoculum for disease. Oospores later germinate to produce either a hyphal tube, which can directly infect a plant, or a germ sporangium, which release zoospores [4]. Therefore, the normal development of sporangia plays key roles in the spread of *P. sojae.* The sporangium of *P. sojae* are multinucleated cells formed as the cytoplasm from the subtending hypha flows into the expanding hyphal apex [5]. After chilling sporangia to approximately 4°C for 30 min, the fusion of cleavage vesicles and release of zoospores occur spontaneously in distilled water. Nuclei within the sporangium organize into a regularly spaced distribution; some membranous elements differentiate in the cytoplasm to produce 10–30 zoospores. Although not as common, sporangia of *P. sojae* can germinate directly and penetrate the host cells at the plant’s root tips. Zoospore motility is important for *P. sojae* to reach its host, chemotaxis and electrotaxis are also essential for locating a favorable infection site. Once a favorable infection site has been located, zoospores encyst, detach their flagella, and secrete adhesive material to become immotile and firmly attached to the plant. Next, the cyst germinates and the germ tube emerges close to the plant and grows chemotropically toward a suitable penetration site. Penetration of periclinal or anticlinal walls may be preceded by the development of appressorium-like swelling of the germ tube. Having penetrated the epidermis, nutrients are acquired through the formation of haustoria [5]. Therefore, the normal sporulation of sporangia with release of normal and functional zoospores is important to complete the *P. sojae* life cycle.

Understanding the molecular mechanisms of sporangial development and release normal zoospores may facilitate development of new strategies for Phytophthora disease control. It is known that MAP kinase signal transduction cascades greatly influence gene expression, metabolism, cell division, cell morphology, and cell survival, and participate in regulating important pathogenic processes in fungi [6]. MAPK is activated by MAPK kinase (MEK or MAPKK), which is activated in turn by MEK kinase (MEKK or MAPKKK). The sequential activation of the MAPK cascade eventually activates transcription factors and the expression of specific sets of genes in response to environmental stimuli [7]. There are direct links between *Arabidopsis* MAP kinase 4 (MPK4) and innate immunity based on releasing transcription factors in the nucleus upon activation [8]. In *Magnaporthe grisea*, the *MST11-MST7-PMK1* MAP kinase (MAPK) cascade is essential for appressorium formation and plant infection [9]. One putative downstream transcription factor regulated by Pmk1 is Mst12 (Ste12 homolog), which is essential for pathogenesis. *MST12* may regulate genes involved in penetration and infectious growth, but another transcription factor(s) must function downstream of *PMK1* to regulate appressorium formation [7]. This MAPK pathway in *M. grisea* has been characterized in detail. MAPK gene homologs have also been characterized in other phytopathogenic fungi. In *Colletotrichum lagenarium*, the conidia of *cmk1* mutants fail to germinate on plant and glass surfaces [10]. In *Bipolaris oryzae*, the rice leaf spot pathogen, *BMK1*, is required for plant infection and conidiation [11]. In the necrotrophic pathogen, *Fusarium graminearum* (wheat scab fungus), *gpmk1* deletion mutants fail to infect roots [12]. In *Botrytis cinerea*, Delta bcsak1 mutants are significantly impaired in vegetative and pathogenic development: they are blocked in conidia formation, and are unable to penetrate unwounded plant tissue [13]. These studies indicate that the MAPK pathway may be conserved in many phytopathogenic fungi and regulate spore sporulation and other plant infection processes.

In contrast, little is known about the MAPK pathway elements or how they are regulated in oomycetes. Bioinformatic searches against the *P. infestans* genome revealed that it contains 15 MAPK, 6 MAP2K, 5 MAP3K, and 4 MAP4K [14]. However, their biological functions are unclear. In *P. sojae*, a stress-activated MAP kinase was identified (named *PsSAK1*) in a previous study, which represents a novel group of MAP kinases [15]. PsSAK1 is currently the only characterized MAP kinase in *Phytophthora*, which is responsible for plant infection processes. It was up-regulated in zoospores, cysts, and during the early infection stages. *PsSAK1*-silenced mutants showed faster encystment, lower germination ratios, longer germ tubes, and colonization defects on both wounded and unwounded soybean leaves. PsSAK1 is an important regulator of zoospore development and pathogenicity in *P. sojae*. To better understand its roles, the transcriptomes of *PsSAK1*-silenced mutant and the corresponding parental control P6497 in two stages were profiled and compared to identify components regulated by PsSAK1. It revealed that several family members of the Myb family were differentially expressed (up or down) in response to the loss of *PsSAK1*. In this study, a Myb transcription factor protein down-regulated in the transcriptomes of *PsSAK1*-silenced mutant is identified and characterized. To elucidate the mechanism of PsSAK1 in *P. sojae* development and pathogenicity, the expression of *Phytophthora sojae* MYB protein 1 (PsMYB1) was silenced through stable transformation in *P. sojae*. The functional analysis of silenced mutants demonstrates that PsMYB1, regulated by PsSAK1, plays key roles in several life-cycle stages that are related to zoospore-mediated plant infection.

## Results

### Identifying Downstream of the Stress-activated MAP Kinase PsSAK1

A stress-activated MAP kinase was indentified and is key regulator of zoospore development and pathogenicity in *P. sojae* [15]. *PsSAK1*-silenced transformants show aberrant zoospore development and less osmotic adaptation. Firstly, zoospore behavior was severely affected by silencing *PsSAK1.* When zoospores were released from sporangia of *PsSAK1*-silenced lines, they appeared as less-active, obese protoplasmic balls, and were not as bean-shaped as zoospores of P6497. Cyst germination was affected by silencing and germ tubes or hyphae of the mutants failed to penetrate host hypocotyls epidermal cells. Secondly, under stress mediated by H_2_O_2_ or Nacl treatment, the expression of *PsSAK1* noticeably increases. Thirdly, *PsSAK1-*silenced strains were growth-impaired in the presence of 0.2 M Nacl compared with the wild-type and control strains. We demonstrate that PsSAK1-mediated pathway exists in *P. sojae* to help regulate the zoosporogenesis, response to stress and infect host.

The 3'-tag digital gene expression (DGE) profiling method, which uses oligo-dT to generate libraries that are enriched in the 3' untranslated regions of polyadenylated mRNAs and produces 21-bp cDNA tags, can measure gene expression level for whole transcriptome [16]. To understand the roles of PsSAK1, we applied DGE profiling for the *PsSAK1*-silenced line T31 using RNAs isolated from cysts and 1.5 h after inoculation onto susceptible soybean leaf tissues (mCY and mIF1.5 h), with the same stages of P6497 wild-type as the parental control (CY and IF1.5 h, [17]). Cysts stage was chosen because of the quicker encystment and a lower germination ratio of *PsSAK1*-silenced transformants. Meanwhile, 1.5 hours after inoculation was chosen as an early time of infection stage based on their phenomenon that could not penetrate the host surface [15]. From the four libraries, 7,493 to 9,846 genes were detected by the DGE profiling tags and a total of 11,081 genes were detected in at least one library (Table S1).

**Figure 1:**
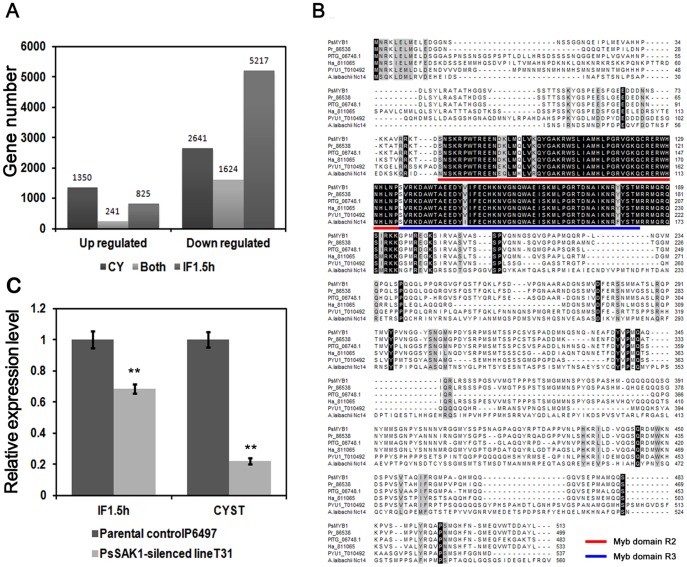
The expression changes and sequence of *PsMYB1*. (**A**) Overview of expression changes during two stages of *PsSAK1*-silenced strain T31. Illustrated are the stages examined (cysts and 1.5 h after inoculation onto susceptible soybean leaf tissues) and number of up- or down-regulated genes during each stage. (**B**) Alignment of *PsMYB1* orthologs. The R2 and R3 Myb DNA-binding domains are underlined by red and blue lines, respectively. The orthologs are from *P. infestans* (PITG_06748.1), *P*. *ramorum* (Pr_86538), *H. arabidopsidis* (Ha_811065), *Pythium ultimum* (PYU1_T010492), and *Albugo laibachii* (A. *laibachii* Nc 14). (**C**) Validation of DGE data by quantitative reverse-transcription polymerase chain reaction (qRT-PCR). *PsMYB1* transcripts in the *PsSAK1*-silenced line are down-regulated dramatically in cysts stage and IF1.5 h stage based on the qRT-PCR. Results of an independent-samples *t-*test for each species. *P<0.05; **P<0.001.

To identify the genes regulated by *PsSAK1*, the gene expression levels between CY-mCY and IF1.5 h-mIF1.5 h were compared respectively to analyze the differentially expressed genes. Two fold was the dividing line with P value≤0.01, employing the False Discovery Rate (FDR) correction. In total, compared to the P6497 wild-type libraries, the numbers of down-regulated (up-regulated) genes were 2,641 (1,350) and 5,217 (825) in mCY and mIF1.5 h, respectively. Moreover, 1,624 and 241 genes were down- and up-regulated in both stages (Figure 1A, Table S1). This suggests that more genes are down-regulated than up-regulated, which also indicates that MAP kinase PsSAK1 activates rather than represses downstream genes when *P. sojae* attaches to the host surface, forms cysts, and penetrates the host surface [15].

Genes that displayed a high response to the loss of *PsSAK1* may play a functional role in disease progression. Functional annotations by BLAST hits, InterPro domains, and gene ontology (GO) terms were collected and are shown in Table S1. The differentially expressed genes encode potential transcription factors, especially Myb, bZIP, zinc finger; potential cellular regulators, such as protein kinases; regulators of RNA synthesis; structural proteins, including putative glycoside hydrolases and proteins involved in cell wall biogenesis; and pathogenic factors, including proteins responsing to stress, and elicitin.

Active MAPKs frequently translocate from the cytoplasm to the nucleus to phosphorylate nuclear targets, transcription factor is a important one [6]. Transcription factors containing two or three imperfect tandem repeats of the Myb DNA-binding domain (named R2R3 and R1R2R3, respectively) regulate important processes in growth and development [18]. Specific processes regulated by Myb proteins in plant include response to environmental stresses in *Arabidopsis* [19], cell and petal morphogenesis in *Antirrhinum majus* [20], drought resistance and salt tolerance in rice [21]. Some lower eukaryotes also express Myb proteins control stress pathway [22].Therefore, we speculate that, Myb proteins may be involved in the stress-associated MAPK PsSAK1pathway in *P. sojae*. To test this hypothesis, by using bioinformatics, annotation of the *P. sojae* genome database, Fungal Transcription Factor Database (FTFD), SMART (http://smart.embl-heidelberg.de), and manual evaluation of gene models, 68 Myb TFs with variable numbers of Myb DNA-binding domains were predicted (Table S2). Most (47) of the proteins have a single Myb domain, whereas the remaining 9, 10, 1, and 1 proteins have two, three, four, and five Myb domains, respectively. On the basis of differential DGE analysis of the *PsSAK1*-silenced line T31, approximately 44 Myb TFs were significantly down-regulated or up-regulated responsing to the loss of *PsSAK1*. These results demonstrate that Myb transcription factor family may play important roles in PsSAK1 pathway (Table S1).

### Identification of the Myb transcription factor PsMYB1

DGE analysis revealed that seven Myb transcription factors were down-regulated significantly in both *PsSAK1* silencing libraries, including *Ps140839* or *PsMYB1* (also named *PsMYB-like 2–7* in Table S2) (140839-incorrect in Table S1). The gene was then cloned from P6497 mycelial cDNA and found previous gene model in the genome database was incorrect, and the corrected *PsMYB1* encodes a 513 amino acid protein with three introns and contains two imperfect tandem repeats of the R2R3 Myb DNA-binding domains (GenBank accession number JX069980) (Figure 1B). Therefore, we updated the gene expression patterns in DGE profiling and found that the expressions of correct gene were down-regulated significantly in two stages (140839-correct in Table S1). To confirm this gene expression patterns derived from DGE profiling and the regulated relationship between *PsMYB1* and *PsSAK1*, we checked the expression level of *PsMYB1* in the cysts or 1.5 hours after inoculation of *PsSAK1*-silenced line T31 by qRT-PCR. The results showed that the transcription patterns of *PsMYB1* were consistent with that in the DGE profiling (Figure 1C). qRT results in combination with the data of DGE profiling indicate that the transcript of *PsMYB1* depend on the presence of a functional PsSAK1 pathway. Each *PsMYB1* ortholog was identified in the sequenced oomycete species including *P. infestans*, *Hyaloperonospora arabidopsidis*, and *Pythium ultimum*. Two were predicted in *P. ramorum* but one sequence possessed an unclear region. Meanwhile, only one gene (CCA17728.1) in *Albugo laibachii*, which is also an oomycete, was identified by BLASTp against the NCBI database with an E-value of 1E-50 as a cutoff (Table S2). Protein sequences alignment among PsMYB1 and its orthologs showed that the R2 and R3 domains had the highest amounts of conservation, whereas the remainder of the gene had relatively low conservation (Figure 1B).

### Expression Profiling and Silencing of *PsMYB1* in *P. sojae* P6497

**Figure 2:**
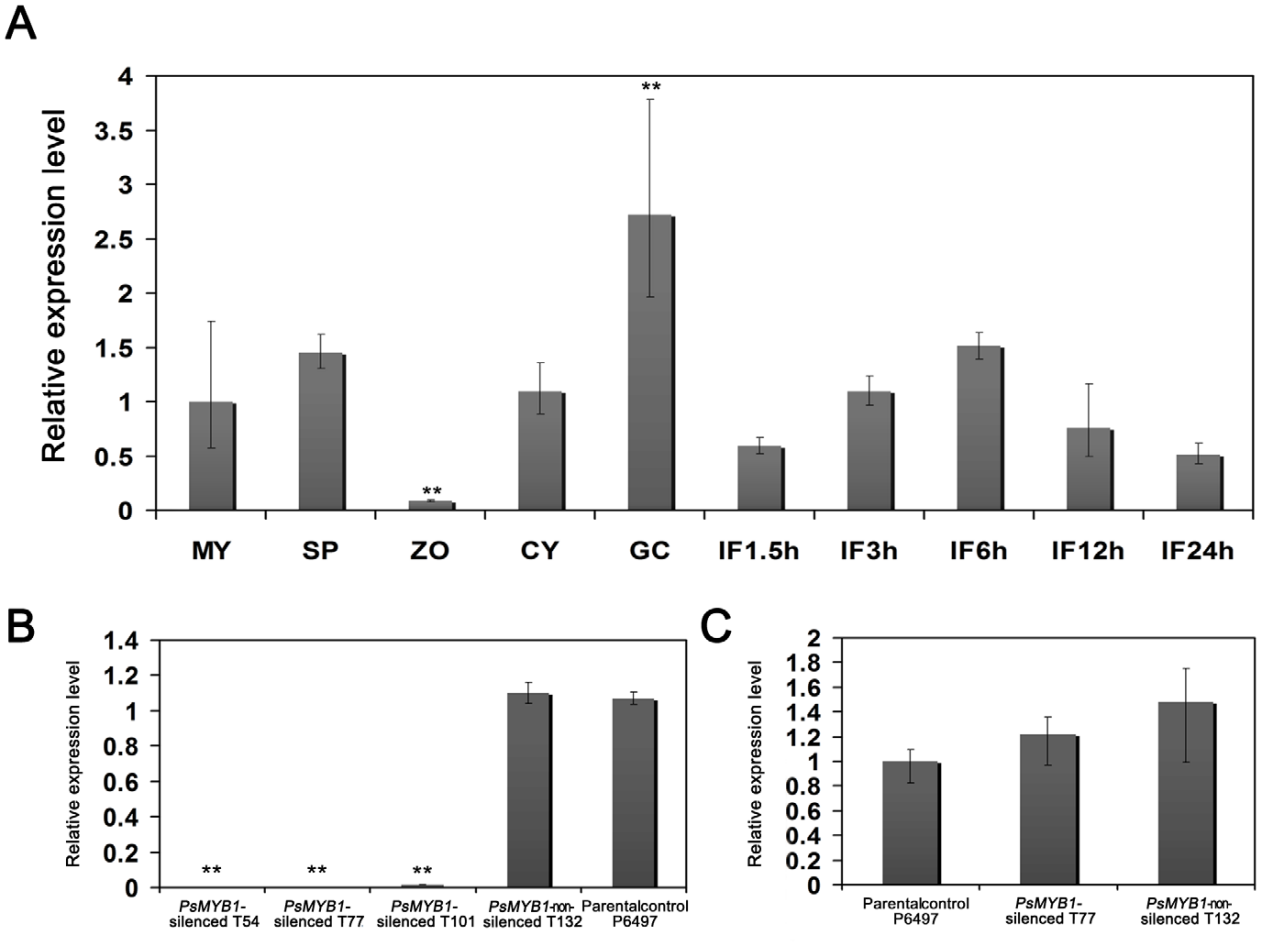
qRT-PCR assay of the *PsMYB1* gene. (**A**) Expression pattern of *PsMYB1* based on qRT-PCR. The ten stages are: mycelia (MY), sporulating hyphae (SP), zoospores (ZO), cysts (CY), germinating cysts (GC), and IF1.5 h to IF24h (samples from 1.5, 3, 6, 12, and 24 h after infection of soybean leaves). (**B**) Expression of *PsMYB1* in strains transformed with extra copies of *PsMYB1*. qRT-PCR was performed using sporangiating hyphae RNAs from wild-type isolate (P6497) and transformants (*PsMYB1* silenced, T54, T77, and T101; *PsMYB1* non-silenced, T132) as initial template. (**C**) *PsSAK1* expression level in the *PsMYB1*-silenced line. qPCR was performed based on the ΔΔCT method. Error bars are calculated from three replicate qRT-PCR assays.

To address the roles of the *PsMYB1* gene, we employed real-time quantitative RT-PCR using RNAs isolated from distinct developmental stages including mycelia, sporulating hyphae, swimming zoospores, cysts and germinating cysts, as well as soybean (Williams) tissues infected with *P. sojae*. *PsMYB1* transcripts were detected in all of the tested libraries but were mostly absent from the zoospores. Expression of *PsMYB1* increased during sporulation, in germinated cysts, and 3–6 h after infection (Figure 2A).

To evaluate the function of *PsMYB1*, the *P. sojae* (P6497) *PsMYB1*-silenced strains were obtained through polyethylene glycol (PEG)-mediated protoplast stable transformation [23]. A total of 152 putative transformants that grew in the presence of Geneticin (50 µg/µl) were selected for further evaluation. Of 152 putative *PsMYB1* transformants, 66 *PsMYB1*-integrated transformants were obtained using genome PCR. Real-time quantitative RT-PCR was used to evaluate the level of *PsMYB1* mRNA accumulation. Finally, three transformants (T54, T77, and T101) failed to produce the given amplicon when the normal number of PCR cycles were applied with sporulating hyphae RNA as initial template (Figure 2B). Therefore, three independent *P. sojae* strains deficient in *PsMYB1* expression were successfully obtained. Furthermore, we have confirmed that lacking *PsMYB1* did not affect the transcript of *PsSAK1* by comparing the transcriptional level of *PsSAK1* with and without functional PsMYB1 (Figure 2C).

### PsMYB1 is Necessary for Normal Asexual Sporangial Development

Phenotypes of the silenced transformants in comparison to controls were evaluated throughout the *P. sojae* life cycle. The controls included the P6497 parental strain (WT), the *PsSAK1*-silenced line (T31), and the transformed strain that failed to exhibit *PsMYB1* silencing (T132). Comparison of the asexual and sexual growth among *PsMYB1*-silenced and non-silenced strains indicated that silencing of *PsMYB1* was not associated with asexual hyphal growth or sexual development (Table 1).

**Table 1:** Comparison of asexual and sexual growth among *PsMYB1*-silenced and non-silenced strains.

Term a	*PsMYB1* silenced			*PsSAK1*-silenced	*PsMYB1* non-silenced	Wild-type
	T54	T77	T101	T31	T132	P6497
Colony diameter(cm) b	4.65±0.20	4.45±0.05	4.97±0.03	4.43±0.22	4.87±0.16	4.87±0.09
Zoospore release(NO.) c	77.67±4.06 _**_	15.33±1.45 _**_	7.48±0.88 _**_	283.00±5.20 _*_	350.42±18.63	425.70±32.55
Encystment (%) d	97.25±0.00 _**_	89.02±0.02 _**_	100.00±0.00 _**_	99.10±0.00 _**_	42.88±0.02 _*_	23.82±0.03
Cyst germination (%) e	69.91±0.02_ *_	26.92±0.02_ **_	44.83±0.02_ **_	44.84±0.03 _**_	95.34±0.01 _*_	86.53±0.02
Oospore(NO.) f	64.11±0.91	49.67±4.91	46.9±0.24	48.65±2.70	42.26±4.18	59.30±6.03

a Strains are the P6497 wild-type (parental) or transformants derived from P6497 (by protoplast transformation). Independent-samples *t-*test between wild-type P6497 and each mutant was performed and marked by "^*^" and "^**^", referring to p<0.05 and p<0.001, respectively. Values in the table are the mean ± standard error.

b Based on 5 days of growth in 10% V8 juice agar medium.

c Number of 5×5 mm colonies releasing zoospores after washing for 24 h at 25°C. The numbers of zoospores in 10 ul of sterile distilled water were counted.

d Percent of encysted zoospores in 50 ul of zoospores suspension after 1 h incubation at 25°C with 80% humidity.

e Percent of cysts forming germ tubes after 2 h incubation with vortexing to induce encystment, based on counting a minimum of 100 cysts from each strain.

f Number of oospores was counted in 10% V8 juice medium for 10 days.

**Figure 3:**
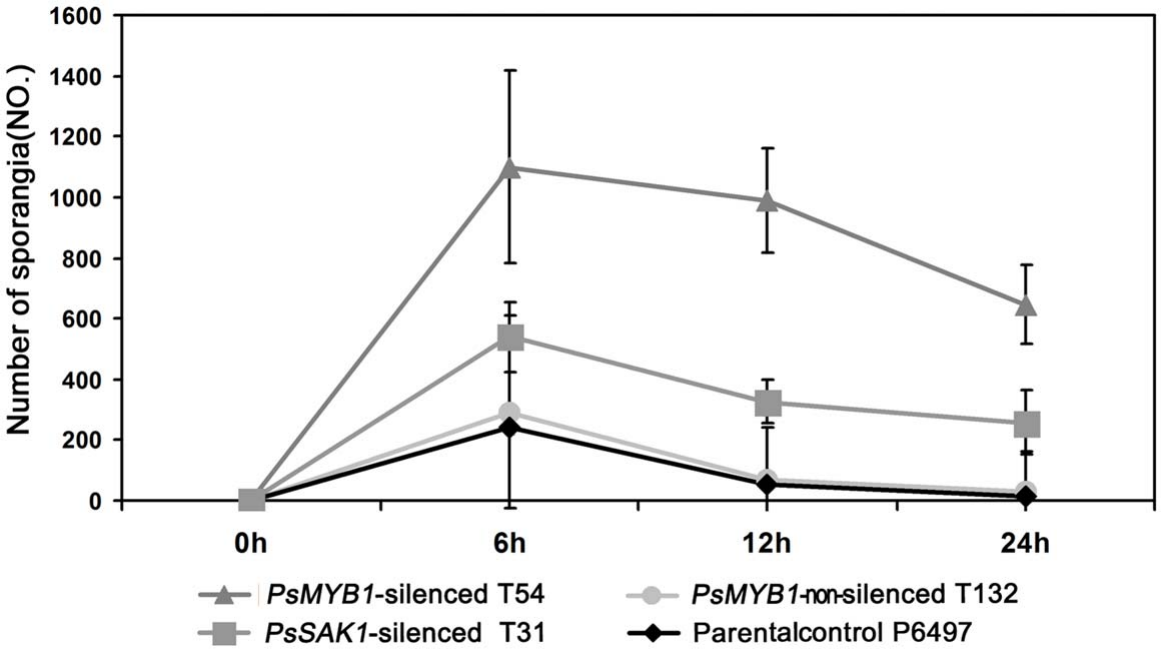
Effect of *PsMYB1* silencing on sporangial development. The numbers of sporangia were counted at 0, 6, 12, and 24 h after inducing *P. sojae* into the sporangial forming stage.

However, the release of zoospores was affected by *PsSAK1* and *PsMYB1* silencing. Instead of releasing swimming zoospores, the majority of sporangia of all three *PsMYB1*-silenced strains germinated directly. During the first 24 h after inducing sporangia production by chilling at 4°C for 30 min and removing to room temperature, we found approximately 59% of sporangia could not release zoospores compared with approximately 5.6% of that in parental control P6497. Over time, the top of the sporangium germinated to form hypha and the cytoplasm flowed from the sporangium to the hyphal tip. After one week, empty sporangia could be observed which consisted only of cell walls. Quantitatively, the numbers of sporangia of strains were counted at 0, 6, 12, and 24 h after washing medium to induce sporangial development (Figure 3). We found that the sporangia of all three controls had releasing zoospores during first 6 h, but not in the three *PsMYB1*-silenced lines. *PsMYB1-*silenced line T54 had the most sporangia (1099.33±317.22) compared to the positive control *PsSAK1*-silenced line T31 (539.33±117.89), negative control *PsMYB1* non-silenced transformant T132 (292.33±14.57), and the parental control P6497 (239.00±23.07). This result is consistent with those of the other two time points. We also found that the parental control wild-type had few sporangia could not release even 24 h after washing with sterile distilled water (13.33±17.09), which is much lower than the number in T54 (647.00±130.03). Thus, the *PsMYB1*-silenced transformant T54 and *PsSAK1*-silenced line T31 produced significantly more directly germinated sporangia than wild-type after 6, 12, and 24 h (T31, p<0.05 and p<0.001 for T54, respectively) (Figure 3). This suggests that normal asexual sporangia development was affected differently by silencing of *PsMYB1* or *PsSAK1*.

### Silencing of *PsMYB1* Impaired Zoosporogenesis and Zoospore Development

**Figure 4:**
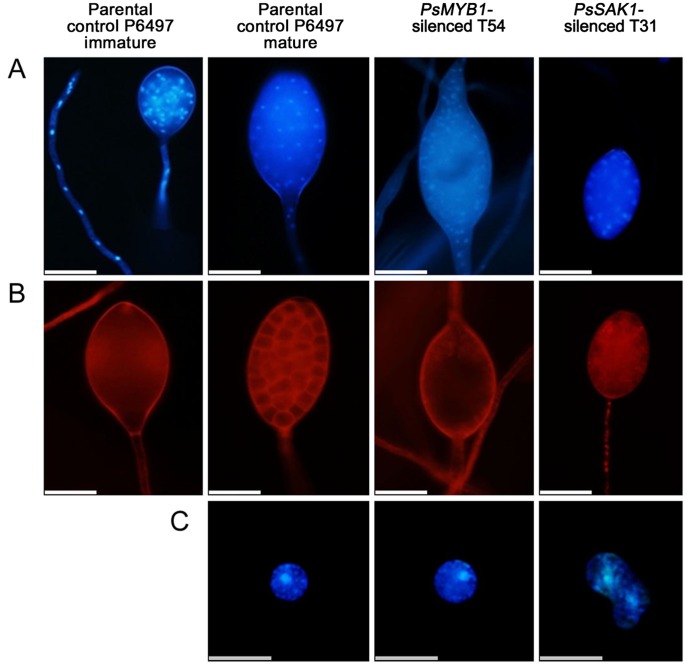
Microscopic observation of the distribution of nuclei and the cleavage system within sporangia or zoospores in *P. sojae.* (**A**) The nuclei distributions in sporangium of early stage of silenced-lines or controls, take parental control P6497 as an example; and the zoosporogenesis stages of P6497, *PsMYB1*-silenced transformant T54, and *PsSAK1*-silenced line T31 were observed with DAPI staining of the nuclei. (**B**) Cleavage-system degeneration during germ tube formation in test strains was seen with FM4-64 to selectively visualize the plasma membrane. The bars at the bottom of (A-B) represent 50 µm. (**C**) Nuclear distribution in zoospores. The bar at the base of the panel represents 20 µm.

In *Phytophthora cinnamomi*, zoosporogenesis involves compartmentalization of the mature multinucleate sporangium into a number of biflagellate and uninucleate zoospores [24]. During direct sporangial germination, the flagella and cleavage system degradation prior to germ tube formation [25]. We hypothesized that PsMYB1 is associated with zoosporogenesis. To explore this hypothesis, specific fluorescent dyes were used to study the effect of PsMYB1 on the organization of internal organelles during sporangial cleavage and zoospore formation. No significant differences in the cellular structure of the controls and *PsMYB1*-silenced sporangia were observed during the early stages. Comparing the silenced strains with controls, sporangia were multinucleated with undifferentiated cytoplasm. Furthermore, the size, form, and structure were similar (immature parental control P6497of Figure 4A and 4B). However, after inducing sporangial cleavage, there were significant differences between sporangia of the controls and the *PsMYB1*-silenced strains. Nuclei within the sporangium of P6497 were regularly spaced; meanwhile, the cytoplasm was differentiated to form an average of 10–30 fully developed zoospores. By contrast, the sporangial cytoplasm of the *PsMYB1*-silenced line T54 remained undifferentiated and the nuclei remained disordered. For the *PsSAK1*-silenced strain T31 (the positive control), nuclei within the sporangium maintained a regularly spaced distribution and the cytoplasm differentiated to form zoospores (mature parental control, T54, T31 of Figure 4A and 4B). We also found that approximately 9% of the zoospores contained two nuclei instead of one, which were not able to swim but instead settled to the bottom of the dish immediately after release from the sporangia (Figure 4C). Our results demonstrate that loss of either *PsSAK1* MAP kinase or *PsMYB1* leads to abnormal *P. sojae* zoosporogenesis.

**Figure 5:**
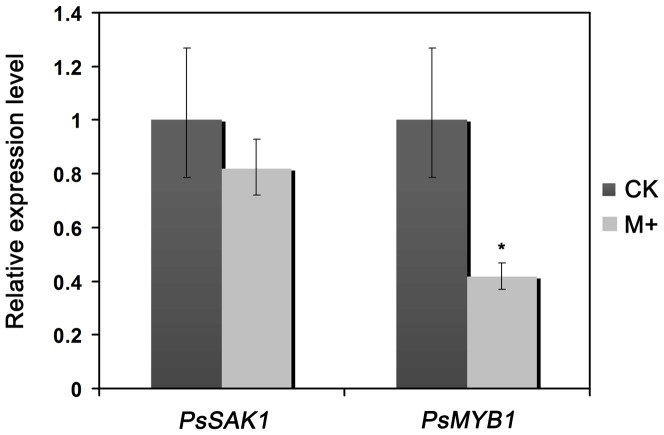
Analysis of *PsMYB1* gene expression during sporangial direct germination. The RNAs of sporulating hyphae were prepared by repeatedly washing 2-day-old hyphae incubated in 10% V8 broth with sterile distilled water and incubating the washed hyphae in the light at 25°C for 4–8 h until sporangia developed on most of the hyphae. "M+", washing the P6497 hyphae with sterile distilled water containing 0.8 M mannitol and induce the sporangia of P6497 to germinate directly. "CK", washing mycelia of the wild-type with sterile distilled water and induce the sporangia of P6497 to germinate indirectly. Evaluating expression of *PsMYB1* in P6497 sporangia of germinated indirectly and that of germinated directly. Expression was measured by quantitative real time PCR and normalized to the level in the sporangia of germinated indirectly of wild-type P6497. Bars indicate standard errors from three independent replicates.

*Phytophthora* sporangia may emit germ tubes directly through the exit pore plug material and sporangia walls when incubated in a nutritive medium with high osmotic pressure [25]. Flagella and cleavage vesicles form during the initial stages of direct germination in the same manner as they do in indirect germination. Later, however, the flagella degenerate and cleavage of the cytoplasm is not complete [26]. The result above demonstrates that PsMYB1 plays key role in regulating zoosporogenesis. Well then, whether the *PsMYB1* would express and execute function or not when the sporangia of *P. sojae* germinate directly. To test the proposition and determine how specific connection between PsMYB1 and sporangia cleaving, we added exogenous mannitol to induce the sporangia of P6497 germinated directly and detected the expression level of *PsMYB1* in this situation, compared with that in sporangia germinated indirectly. 0.8M mannitol was added to sterile distilled water which was used to wash the 2-day-old wild-type hyphae to induce the sporangia germinated directly, using sterile distilled water alone to induce the sporangia of P6497 germinated indirectly. After 5 h we observed abundant formation of sporangia in both treatments. RNAs were isolated to examine the gene expression levels of *PsMYB1*, using *PsSAK1* expression as a positive control. As shown in Figure 5, the abundance of *PsMYB1* transcript in the sporangia exposed to 0.8 M mannitol was significantly lower than in the treatment by ddH_2_O_2_ only, with down-regulation >2.5-fold. That suggests the transcription of *PsMYB1* is suppressed in direct sporangial germination stage of *P. sojae*. The result clarified the connection between PsMYB1 and sporangia cleaving from the contrary aspect.

Although most sporangia directly germinated germ tubes, there were also few sporulate zoospores. This was quantitatively assayed for each transformant by counting the zoospores released from a 5×5 mm colony. The amounts of zoospores released from the silenced strains (T54: 77.67±4.06, T77: 15.33±1.45, T101: 7.48±0.88) were significantly less than those released from the controls (T132: 350.42±18.63 and P6497: 425.70±32.55) and T31 (283.00±5.20) based on an independent-samples *t-*test (P<0.001) (Table 1).

The time span between zoospore release and encystment was significantly affected by silencing. Quantitatively, the silenced strains showed more rapid encystment than the control strains. The mean percent of zoospores that changed into cysts in the mutant strains during 1 h after release were 97.25±0.00 (T54) and 89.02±0.02 (T77) compared with 48.22±0.02 in the negative control strain T132 and 23.82±0.03 in the parental control P6497, which represents a significant difference based on an independent-samples *t-*test for each species (P<0.001). Especially for T101, all zoospores formed cysts within 0.5 h after releasing from the sporangia (Table 1). By analyzing these zoospores using microscopy, we found that zoospores released from the abnormal sporangia could not swim efficiently but instead remained near their release point, which was consistent with the *PsSAK1*-silenced line.

Furthermore, the cyst germination rates were lower in the mutants than in the negative control strains (Table 1). Although the average rate of cyst germination of each *PsMYB1*-silenced strain varied under the same conditions, there was a clear difference between *PsMYB1*-silenced lines and the negative control strains; e.g., mutants T77 (26.92±0.02), T101 (44.83±0.02), and T54 (69.91±0.02) compared with T132 (95.34±0.01) and P6497 (86.53±0.02). This suggests that the number of germinated cysts were reduced significantly by silencing of *PsMYB1* based on an independent-samples *t*-test for each species (P<0.001). Meanwhile, the positive control *PsSAK1*-silenced line T31 had a similar phenotype.

Overall, these results demonstrate that PsMYB1 is regulated by PsSAK1 and affects zoospore viability.

### PsMYB1 is Essential for Zoospore-mediated Plant Infection

**Figure 6:**
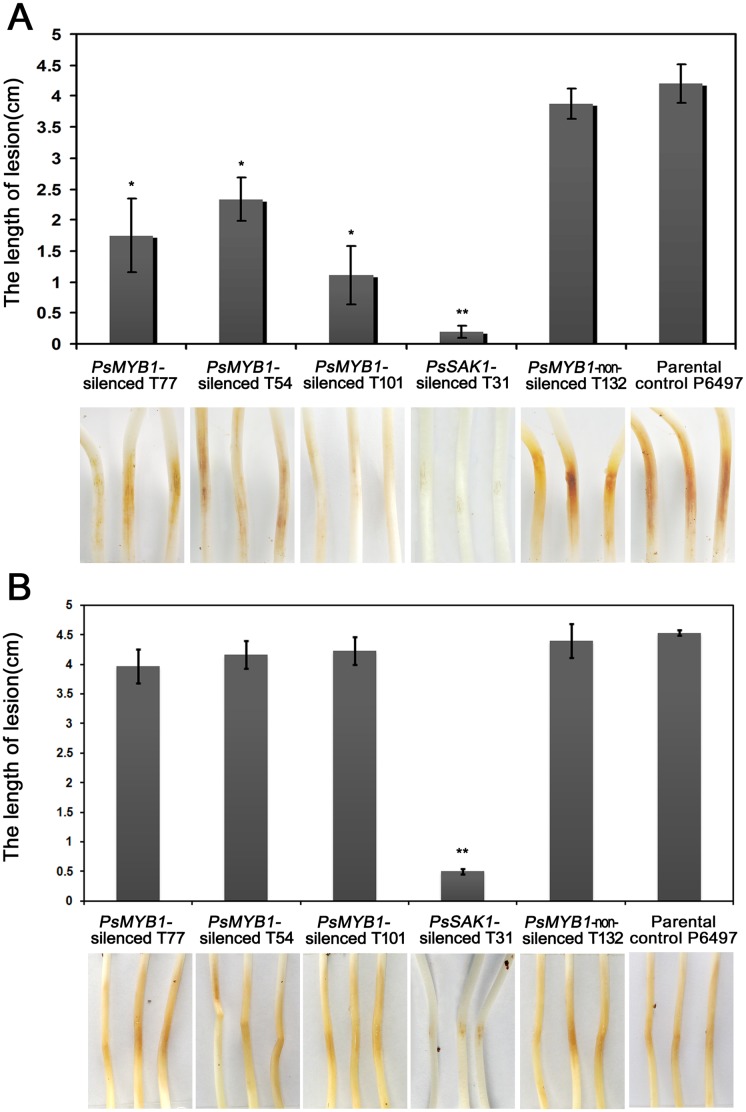
Pathogenicity assay. (**A**) Disease lesion lengths in etiolated soybean hypocotyls infected with zoospores of wild-type and transformed strains of *P. sojae*, 48 h after inoculation. (**B**) Disease lesion lengths in etiolated soybean hypocotyls infected with hyphal tip plugs of wild-type and transformed strains of *P. sojae*, 48 h after inoculation. Results of an independent-samples *t-*test for each species. *P<0.05; **P<0.001. Results of statistical comparison between each mutant and P6497 are in the test. The large water-soaked lesions coating the surface of the etiolated seedling represent a successful infection. The photos were taken after incubation at 25°C for 48 h in the dark.

Silencing also affected zoospore-mediated pathogenicity, as would be expected for strains defective in zoospore release and cyst germination. To test the pathogenicity, we independently applied mycelia and zoospore suspensions of three *PsMYB1*-silenced transformants (T77, T54, and T101), one *PsSAK1*-silenced line (T31), *PsMYB1* non-silenced transformant (CK: T132), and the parental control P6497 on etiolated hypocotyls at 25°C to enhance disease progression. In the zoospore assay, all silenced transformants failed to infect based on the shorter lesion lengths compared to the wild-type strain P6497 (Figure 6A). We hypothesized that this was due to the reduced cyst germination of *PsMYB1*-silenced transformants. That was confirmed by comparing the cyst germination ratio and virulence on soybean plants (Table 1 and Figure 6A). However, when inoculated with hyphal tip plugs, both negative control strains (T132 and P6497) and *PsMYB1*-silenced transformants caused spreading water-soaked lesions that are characteristic of virulent (Figure 6B).

## Discussion

Our results demonstrate that PsSAK1 elicits a robust transcriptional response, part of which is functionally important for zoospore development. Loss of *PsSAK1* induced (repressed) the expression of 2,641(1,350) genes during the cysts stage and 5,217 (825) genes in the IF1.5 h library in *P. sojae* compared to the control. Several family members of the Myb transcription factor were also differentially expressed (up or down) in response to the loss of *PsSAK1*. DGE profiling revealed a R2R3-type Myb transcription factor named *PsMYB1* was down-regulated significantly in two *PsSAK1* silencing libraries. *PsMYB1* transcripts increased during sporulating hyphae, germinated cysts and 3–6 h after infection, but were mostly absent during the zoospores stage. Loss of *PsMYB1* resulted in the sporangia of mutants remaining undifferentiated with disordered nuclei after inducing zoospore formation for 6–8 h when that of the parental control P6497 had released numerous zoospores. These results demonstrate for the first time that sporangial development and release of normal zoospores in *P. sojae* depend on the PsMYB1 pathway, regulated by PsSAK1.

The Myb transcription factor plays an important role in zoospore development of oomycetes. This is supported by the function of *PsMYB1* orthologs in other oomycetes. In *P. infestans*, semi-quantitative RT-PCR was performed against the *PsMYB1*ortholog, *PiMyb2R3* (PITG_06748.1), during asexual sporulation or germination. *PiMyb2R3* transcripts persisted in sporangia, during zoosporogenesis (i.e., cleaving sporangia), and in swimming zoospores, but decreased during the germinated cysts stage. When sporangia were placed in broth media to induce the production of mycelia from sporangia, *PiMybR2R3* mRNA disappeared. This is consistent with the sporangial direct germination phenotype of the *PsMYB1*-silenced line. For *H. arabidopsidis*, which lacks the zoospore stage, there is only 14 Myb transcription factors left in the genome, compared with 68 in *P. sojae*. *PsMYB1* expressed at a higher level in sporangia stage, which is supported by a prior microarray study showing that most of the zoospore-specific genes in *P. infestans* were induced during sporulation. Meanwhile, most sporulation-induced genes are absent from *H. arabidopsidis* compared to *P. infestans*, which is consistent with the inability of *H. arabidopsidis* to produce zoospores [18]. In this study, we focused on the biological function of *PsMYB1* based on its silencing and the analysis of its gene expression patterns. From the findings that inhibition of *PsSAK1* resulted in defective zoospores and loss of *PsMYB1* resulted in malfunctions of zoospore development, we identified a role for the *PsMYB1*, regulated by the MAP kinase *PsSAK1*, in zoospore development.

A challenging step in further understanding the MAPK pathway is the identification of downstream genes that are regulated by MAPK. Here, the analysis of 3′ tag digital gene expression profiling using a *PsSAK1* mutant obtained by PEG-mediated protoplast stable transformation led to the identification of a downstream gene of the MAPK PsSAK1 pathway, *PsMYB1*. *PsMYB1* is transcriptionally suppressed after loss of *PsSAK1*, and silencing of *PsMYB1* does not affect the transcriptional level of *PsSAK1*, which suggests partially that PsMYB1 is positively regulated by PsSAK1 (Figure 1C and Figure 2C). These data suggest that activation of the MAPK pathway leads directly or indirectly to increase production of PsMYB1 that in turn leads to zoosporogenesis and development of normal zoospores. Silencing of *PsMYB1* results in undifferentiated sporangia that cannot form and release zoospores. Compared with *PsSAK1*-silenced lines which release less-active zoospores and contain obese protoplasmic balls, *PsMYB1*-silenced transformants display stronger malfunctions. However, loss of *PsMYB1* has a weaker effect on virulence compared with the *PsSAK1*-silenced line. Thus, it is possible that the PsSAK1 MAPK pathway also regulates other genes which are important pathogenic factors in *P. sojae*. It is also possible that there is crosstalk between two pathways. Like in *Cryptococcus neoformans*, inhibition of calcineurin induces the phosphorylation of Mpk1 and the induction of *FKS1*, suggestive of an interplay between the CWI pathway and calcineurin [27]. Similar to these mechanisms, it is possible that PsMYB1 is activated by another pathway(s) when PsSAK1 is inhibited. The DGE profiling analysis supports this concept. Loss of *PsSAK*1 up-regulated 1,350 and 825 genes in the cyst and IF1.5 h stages, respectively.

Our results demonstrate that PsMYB1, regulated by PsSAK1, is functionally important for zoospore development and release. We note that the phenotype of inhibition of zoospore development and release, and specifically that of direct germination can be recreated via manipulation of exogenous calcium. In *P. infestans*, inhibitors of calcium pathways strongly inhibited zoospore release [28]. It was found that in *Phytophthora cinnamomi*, a longer lasting rise in Ca^2+^ is required during cytoplasmic cleavage to regulate cytokinesis. Conversely, the calcium channel blockers were also found to inhibit zoosporogenesis and encystment [29]. In *Phytophthora parasitica*, Ca^2+^ treatments affected the production of zoospores from sporangia at two different stages: during cleavage of the sporangium protoplasm into uninucleate cells and at the stage when zoospores were released by dissolution of the sporangium papillum [30]. Here, we note that the *PsSAK1*-silenced lines and *PsMYB1*-silenced strains display the similar phenotype. Thus it is possible that PsSAK1 mediates its affects by affecting proteins which mediate signaling of calcium, and vice versa. We intend to further explore this hypothesis in our future work.

We have found that PsMYB1, regulated by PsSAK1, plays key roles in zoospore development, but how PsSAK1 regulated PsMYB1 to affect zoosporogenesis is not clear. As is known, transferring sporangia to pure water initiates zoospore release after a short time, which demonstrates that very few conditions are need for triggering this process. The most important preconditions for zoospore release are a turgor pressure in the sporangium high enough to initiate the process and an osmotic gradient between sporangium and external medium high enough to allow a significant water uptake into the sporangium [25]. In the previous study, we found that PsSAK1 is required for *P. sojae* response to extracellular osmotic stimuli because the expression of *PsSAK1* noticeably increases under stress mediated by H_2_O_2_ or Nacl treatment, and loss of *PsSAK1* lead to sensitive to 0.2M Nacl [15]. Perhaps, loss of *PsSAK1*, *P. sojae* could not response the change of extracellular osmotic pressure. Thus, there may be not an osmotic gradient between sporangium and external medium, and the turgor pressure of sporangium is not high enough to initiate the PsMYB1 in regulating zoosporogenesis. In near-isotonic solutions, the cleavage of zoospores may be incomplete.

Our DGE profiling and silencing lines data indicate that PsMYB1, regulated by MAP kinase PsSAK1, is responsible for zoosporogenesis and the development of normal zoospores, and affects zoospore-mediated plant infection. These results demonstrate for the first time that PsSAK1 pathway plays an important role in *P. sojae* zoospores development by regulating PsMYB1.Further studies on PsMYB1 in *P. sojae* will yield important insights into the regulation mechanism of PsMYB1 and increase our understanding of its signaling pathway.

## Materials and Methods

### Preparation of *Phytophthora sojae* Isolates, Plant Materials, and Virulence Tests

*Phytophthora sojae* P6497 was from Brett Tyler (Oregon State University, USA). *P. sojae* isolates were maintained on 10% V8 agar media at 25°C in the dark [31]. To identify the asexual and sexual growth, each strain of *P. sojae* was grown on plates (9 cm in diameter) containing 10 ml V8 juice agar at 25°C. Individual agar disks (5 mm in diameter) were removed from the edge of an actively growing culture into the center of the plate. After 5 days, the colony diameter was measured. Zoospores were produced by placing three V8 agar disks (5 mm in diameter) from the edge of an actively growing culture of each strain into a plastic plate containing 150 µl of 10% V8 juice for one disk, and then incubating these dishes in darkness at 25°C for 24 h. After using 150 µl of sterile distilled water to wash the mycelium three times, another 150 µl of sterile distilled water was added to induce the sporangia in light at 25°C for 24 h. Next, the agar disks were removed and the zoospores suspension was measured. We manually counted the number of zoospores in 10 µl of zoospores suspension. We analyzed zoospore encystment and the germination of cysts as described by Hua [23]. Five individual agar disks (5 mm in diameter) of test strains were placed into glass plates (9 cm in diameter) containing 10 ml of 10% V8 juice broth in darkness at 25°C for 10 days to induce oospore formation. Three replicates were made for each transformant, and every replicate contained three culture plates. We mixed the culture of three plates and added it to a homogenate cup for 5 min (3000 r/min), after which we counted the number of oospores in the 10 µl of homogenate. All determinations of each strain were performed in triplicate which contained three duplications. We took the mean of the three duplications as the result of one replicate. For infection assays, the hypocotyls of soybean (Glycine max) cultivar Williams (rps) were used to evaluate the virulence of *P. sojae* cultures. Quantitative virulence of strains was measured using a lesion length assay, as described previously [32]. Etiolated seedlings (grown at 25°C in the dark at 80% humidity for 3–4 days) were inoculated 2 cm below the beginning of the root zone for the wild-type and the transformants. Each was spot incubated with hyphal tip plugs or 100 zoospores, sealed in plastic bags, and incubated at 25°C in the dark for 2 days. The disease lesion length from the inoculation point up and down the hypocotyls was then measured. Each treatment contained at least 5 plants. All treatments were replicated in triplicate independently. All statistical analyses were performed with PASW statistics 18 (SPSS Inc., USA).

### Digital Gene Expression Profiling and Bioinformatics Analysis

The preparation of biological material, libraries, and RNA sequencing was conducted according to published methods [17]. For the wild-type stages (CY and IF1.5 h), we used the raw tags and the calculated gene expression levels, which were published previously [17]. The sequences of *PsMYB1* orthologs from different organisms were obtained from the NCBI, Broad Institute databases, and DOE Joint Genome Institute, using the BLASTp program. Multiple sequence alignments were generated and drawn by BioEdit 7.0.1.

### Nucleic Acid Manipulation

Genomic DNA extraction was performed from hyphae grown in 10% V8 liquid medium, as described previously [33]. Total RNA was isolated using a PureLink RNA Mini Kit (Invitrogen, USA) following the manufacturer’s protocol. To confirm the relative abundance of *PsMYB1* transcripts derived from the DGE libraries and the *PsMYB1* expression patterns during 10 developmental and infection stages of *P. sojae*, the total RNA samples were prepared according to published methods [17].

### *P. sojae* Transformation, Quantitative RT-PCR, and Gene Expression Analysis

For *PsMYB1* silencing, *Phytophthora sojae* transformation plasmid pHam*PsMYB1* (part sequence of *PsMYB1*,1084 bp of negative strand, driven by Ham34 promotor) was contructed as follows. The NptII gene of pHAMT35N was replaced with 1084 bp of *PsMYB1* [34]. The *PsMYB1*-silencing sequence was confirmed by EST, using the primers MYB1-2F,5′ -GGCAACAGCAACAGCAGCGG-3′ and MYB1-2R,5′-TACGGGGAGTTCATCATGCCC-3′. The transformation experiments were performed as described previously [35] except for the use of two different concentrations of G418 before transfer to the V8 agar medium with 50 µg/ml G418. After the protoplast overnight regeneration, we suspended them in liquid Pea/0.5 M Mannitol (PM) media (40°C) containing 30 µg/ml G418 and then plated the suspension. When visible colonies were observed after 2 to 3 days’ incubation at 25°C, we overlaid the plate with Agar Pea/0.5 M Mannitol (PM) media (40°C) containing 50 µg/ml G418 and plated again at 25°C for 2–3 days. Next, the transformants were propagated on V8 agar with 50 µg/ml G418 at 25°C. This modification decreases false positive rates. Of these, approximately 33% were found to contain the transgene. Putative *P. sojae PsMYB1*-silenced lines were screened as follows. First, genomic PCR screening of all transformants was performed with oligonucleotides HMF (5′-TTCTCCTTTTCACTCTCACG-3′) and HMR (5′-AGACACAAAATCTGCAACTTC-3′), which are primers for the Ham34 promoter and terminator, respectively. Next, to investigate the silencing efficiency of the *PsMYB1* transgene in the transformants, total RNAs of positive inserted transformants in the sporulating hyphae stage were extracted and isolated. qRT-PCR was performed as described previously [36], except using oligonucleotides MYB1qRTF(5′-GAACCAGAGCAACCAGAATG-3′) and MYB1qRTR (5′- TACGGGGAGTTCATCATGCC-3′).

### Microscopy

For cell biology assays, sporangium and zoospores were stained with the blue-fluorescent DAPI nucleic acid stain 4′, 6-diamidino-2-phenylindole, dilactate (Invitrogen), to visualize the distribution of nuclei within sporangium and the number of nuclei in zoospores of the transformants or parental control P6497. We equilibrated the sporulating hyphae of different stages briefly with phosphate-buffered saline (PBS) and diluted the DAPI stock solution to 300 nM in PBS. Approximately 300 µl of this dilute DAPI staining solution was added to the coverslip preparation. After incubating for 1–5 minutes, we rinsed the sample several times in PBS and then viewed the sample using an Olympus 1X71inverted microscope at 330–385 nm. Red-fluorescent FM® 4–64 dye, which selectively visualizes the plasma membrane, was used to monitor the cleavage system in sporangia. First, we prepared the samples as described above. At the same time, a working staining solution of 5 µg/ml dye in ice-cold HBSS was prepared, which was kept on ice. Next, the coverslip with the sample was immersed in the staining solution on ice. After 1 minute, we removed the coverslip from the staining solution and imaged the sample without washing. The filter sets used were 510–550 nm.

To determine differences in sporangial development between P6497 and the silenced lines intuitively and quantitatively, 2×2 mm hyphal tip plugs of stains were cut to inoculate 150 µl of sterile clarified 10% V8 juice. Stationary mycelia cultures were incubated at 25°C in the dark for 24 h. Next, we washed the hyphae with sterile distilled water three times and incubated them in the light at 25°C with 100 µl of sterile distilled water. Samples were taken after 0, 6, 12, and 24 h incubations, and pictures were taken using a stereo microscopic Olympus SZX10. Subsequently, the number of sporangia that did not release was counted under the microscope. This assay was repeated at least three times, and the statistical analyses were performed with PASW statistics 18 (SPSS Inc., USA).

## Supporting Information

Table S1 **Gene expression profiling in two stages for the** ***PsSAK1*** **-silenced transformant T31 vs.** ***P. sojae*** **P6497.** *PsSAK1* and *PsMYB1* were labeled by red, and the other family members of Myb were labeled by blue color.
(XLS)

Table S2 **The predicted Myb TF family genes.**
(XLS)
